# Cognitive training based on metamemory and mental images: Follow-up evaluation and booster training effects

**DOI:** 10.1590/S1980-57642011DN05010009

**Published:** 2011

**Authors:** Flávia Ogava Aramaki, Mônica Sanches Yassuda

**Affiliations:** 1,2 Escola de Artes, Ciências e Humanidades da Universidade de São Paulo, São Paulo SP, Brazil.

**Keywords:** cognitive training, imagery, self-efficacy, metamemory, seniors, aging

## Abstract

In the second semester of 2008, 37 seniors participated in five cognitive training sessions based on creation of imagery and modification of self-efficacy for memory tasks.

**Objective:**

To carry out a follow-up evaluation after 18 months in order to detect possible maintenance of gains reported in the first post-test, namely, in measures of self-efficacy and episodic memory, and to evaluate the impact of a training booster, that is, test whether there are additional gains when training is offered for the second time to the same participants.

**Methods:**

16 older adults agreed to participate in five training sessions for the second time. Participants were evaluated with the Mini Mental Status Examination - MMSE, the Geriatric Depression Scale - GDS, the Brief Cognitive Screening Battery - BCSB (naming and memorization of 10 pictures, animal category verbal fluency test, the Clock Drawing Test - CDT), the Story subtest from the Rivermead Behavioural Memory Test - RBMT, the Memory Complaint Questionnaire - MAC-Q, and the Picture and Story domains from the Memory Self-Efficacy Questionnaire - MSEQ used to evaluate the effectiveness of the first intervention.

**Results:**

This study reports the maintenance of the effects generated by the original training conducted in 2008, and follow-up evaluations detected the presence of potential additional gains in some aspects of memory.

**Conclusions:**

Training boosters may help maintain cognitive stability in adulthood and old age.

## Abstract (pt)

No segundo semestre de 2008, 37 idosos participaram de treino cognitivo de cinco sessões baseado na criação de imagens mentais e na alteração da auto-eficácia para tarefas de memória.

**Objetivo:**

Realizar a avaliação de seguimento, após 18 meses, com o intuito de detectar possível manutenção dos ganhos documentados no primeiro pós teste, a saber, em medidas de auto-eficácia e memória episódica; e avaliar o impacto de reforço de treino cognitivo, isto é, avaliar se ocorrem ganhos adicionais quando o treino é oferecido pela segunda vez aos mesmos participantes.

**Métodos:**

16 adultos mais velhos ou idosos aceitaram participar de cinco sessões de treino pela segunda vez. Os participantes foram avaliados com o Mini Exame do Estado Mental - MEEM, a Escala de Depressão Geriátrica - EDG, a Bateria Breve de Rastreio Cognitivo - BBRC (nomeação e memorização de 10 figuras, fluência verbal animais, Teste do Desenho do Relógio), o subteste História do Teste Comportamental de Memória de Rivermead - RBMT, o Questionário de Queixas de Memória - MAC-Q, os domínios Figura e História do Questionário de Auto-Eficácia para Memória - MSEQ, que foram utilizados na avaliação da eficácia da primeira intervenção.

**Resultados:**

O presente estudo documentou a manutenção dos efeitos gerados pelo treino original realizado em 2008, e possíveis ganhos adicionais em alguns aspectos da memória após a segunda intervenção.

**Conclusões:**

O reforço de treino cognitivo pode ajudar a manter a estabilidade do desempenho cognitivo na vida adulta e velhice.

Aging populations are a worldwide phenomenon that is becoming increasingly more frequent. The combined fall in both fecundity and death rates have led to a change in the age structure of the Brazilian population, with a relative decrease in the younger populations and an increase in the proportion of older adults.^1^

The aging process is associated to a natural gradual decline in some cognitive functions, dependent on neurological processes. These processes in turn, may undergo changes with time. The natural decline in some aspects of memory, in the absence of disease, does not compromise the autonomy of older adults, since the majority of elderly retain sufficient cognitive skills to remain independent.^2^

However, the memory subsystems are not affected equally. Decline in episodic memory, involved in storing facts, is greater compared to semantic memory which is involved in memorizing linguistic content.^3^ Explicit memory, also known as conscious memory, appears to be more sensitive than implicit memory, or unconscious memory, to the effects of aging.

Evidence in the literature indicates that memory can be optimized in old age by maintenance or improvement in performance through cognitive interventions. Studies available on cognitive training are promising with regard to natural aging-associated losses, in that aging is a dynamic process which may be associated with losses, stability and growth.^2^

However, it is known that the elderly tend to hold more negative beliefs about memory compared to younger individuals. The term metamemory defines a construct encompassing knowledge, perceptions and beliefs about memory itself and about memory systems in general.^4^ Metamemory also encompasses self-efficacy, which is the perceived ability to carry our memory tasks, as well as performance expectations and affects on memory^5^. The variables related to metamemory can influence performance on cognitive activities.^6^

Interventions aimed at changing negative beliefs about memory and teaching the use of mnemonic strategies can improve objective and subjective memory at the same time. However, the results of these hybrid studies remain controversial.^7^

According to the literature, cognitive training can prevent or attenuate age-associated memory losses.^8^ In addition, these studies have significant implications for the routine clinical practice of gerontologists since, according to a longitudinal study examining factors influencing risk of death in old age, the maintenance of memory and successful aging are interconnected, given that memory is associated to individual autonomy and consequently to quality of life among the elderly.^9^ This is compounded with the fact that many older adults are concerned about their memory and their performance in cognitive activities, making cognitive plasticity an important field of study in gerontology.

Besides gains in objective performance on memory tasks, possible secondary benefits of training have also been reported in the literature. These include greater processing speed, improved concentration and attention, a lower level of anxiety, creation of personal strategies, increased social contact, and consequently lower degrees of social isolation and depression.^10^

According to Brazilian studies conducted in recent years, training involving five or more sessions focused on only a few memory strategies tend to produce the best results.^11-14^ The interventions studied to date also seem to produce significant results in older adults with low schooling^14^. Although a few follow-up and retraining studies have been conducted in other countries, studies performed in Brazil have not included follow-up assessments, while the efficacy of booster training some months after completion of the original intervention has yet to be tested.

The aim of the present study was to assess the maintenance of gains reported after an intervention consisting of five sessions^13^ 18 months after its conclusion, namely in measures of self-efficacy and episodic memory. Additionally, the study sought to assess the impact of cognitive booster training, i.e. to evaluate additional gains when the training intervention is given for a second time to the same participants.

## Methods

### Participants

This study involved 16 older adults enrolled with a University open to the third age, within a public University, who had taken part in five-session memory training in 2008. The sample comprised 15 women and one man, with a mean age of 65.6 years (SD=5.6ys) and a mean of 9.5 years of schooling (SD=3.9ys). Non-returners gave a variety of reasons including health grounds, caregiver obligations, or having another commitment which clashed with the times of the training sessions.

### Instruments

All subjects completed an initial assessment (identifying the pre and post assessments performed in 2008) consisting of a sociodemographic questionnaire, a question on predicted performance: “If someone showed you a sheet containing 10 different figures and you had 30 seconds to memorize them, how many figures do you think you’d be able to remember?”, the Mini Mental State Exam - MMSE^15^, Geriatric Depression Scale with 15 questions - GDS^16^, the Brief Cognitive Screening Battery - BCSB^17^ which includes Naming and memorization of 10 pictures; animal category verbal fluency test, the Clock Drawing Test - CDT, two different versions of the Story subtest from the Rivermead Behavioural Memory Test - RBMT^18^, the Memory Complaint Questionnaire - MAC-Q^19,20^, and finally the Picture and Story domains from the Memory Self-Efficacy Questionnaire - MSEQ.^21^ For a more detailed description of these instruments see.^13^

None of the participants presented scores suggestive of dementia or of depression on the MMSE and GDS-15, respectively.

### Procedures

The participants of the first intervention were invited to take part in a booster training intervention, which consisted of a repeat of the 5 sessions given in 2008. Subjects were assessed before and after doing the booster. The pre and post test data of the new intervention were compared against the pre and post data from the first intervention. The data on those taking part in the booster training were compared to the data they reported in the 2008 intervention.

The 16 participants were divided into two groups to allow the participants to glean greater benefit from the sessions. Each of the five training sessions included a 45-minute psycho-educative intervention aimed at changing negative beliefs about memory in aging and at increasing participants’ knowledge on the functioning of memory. Aspects of memory which undergo only slight age-associated changes were discussed at each session, such as semantic and implicit memory. At all sessions, participants were asked to report memory successes, i.e. occasions on which they managed to remember important information. Subsequently, participants spent 45 minutes learning how to use mental images for memorizing words, phrases and short stories, in this order, whereby the complexity of memorization tasks was increased every session. During this activity, the participants were encouraged to close their eyes and visualize the items to be memorized, which may appear to be related to each other. Vivid images, which could include a touch of humor or unusual content, were encouraged. The sessions were held twice a week.

### Ethical aspects

This study was approved by the Research Ethics Committee of the Psychology Institute of the University of São Paulo. Prior to commencing the assessment, all participants of the study signed an Informed and Free Consent Term. After finishing the cognitive training in 2008 and 2010, the participants received feedback on their performance during training, and were given a certificate of participation.

### Statistical analyses

The data collected were analysed using version 17.0 of the SPPS package. The Kolmogorov-Smirnov test was used to verify whether variables possessed a normal distribution. Given that data was found to have a normal distribution, parametric tests were used. The performance of the sample on pre and post tests during the first and second intervention was compared using Student’s t test for paired samples. Performance on the first post test was compared with performance on the second pre-test to verify maintenance of gains created during the first intervention. This comparison was also carried out using Student’s t test for paired samples. A level of statistical significance of p<0.05was adopted.

## Results

Table 1 shows the mean for cognitive variables on the pre and post tests for the first (2008) and second (2010) interventions. The data in Table 1 reveal that the participants had greater performance on the post test after the first intervention for Incidental Memory, Delayed Memory, and on the story subtest of the Rivermead for delayed recall. In addition, participants scored higher (marginally significant, p value between 0.05 and 0.09) in the post test on the MMSE and on immediate recall in the story from the Rivermead test.

**Table 1:** Mean pre- and post-test performance in 2008 and 2010.

	Pre-test 1 (2008)	Post-test 1 (2008)	p value*	Pre-test 2 (2010)	Post-test 2 (2010)	p value*
Prediction	5.8 (2.2)	6.6 (2.3)	.228	7.4 (1.3)	7.8 (1.1)	.456
MMSE	27.8 (1.7)	28.5 (1.3)	.075	28.9 (1)	29.7 (0.6)	.003^a^
MACQ	25.2 (2.9)	24.1 (2.5)	.171	24.4 (3.1)	22.7 (3.5)	.029^a^
Verbal fluency	16.5 (3.1)	16.9 (3)	.485	17 (3.4)	18.5 (3.9)	.332
Incidental memory	5.8 (1.2)	7.4 (1.4)	.005^a^	7.4 (1.2)	8.6 (1.4)	.013^a^
Immediate memory	9.0 (1)	9.1 (1.2)	.456	9.1 (1.2)	9.6 (0.6)	.070
Delayed memory	8.0 (1.5)	8.9 (1.1)	.011^a^	9 (1.1)	9.6 (0.6)	.013^a^
RBMT story immediate	6.7 (1.5)	8.3 (2.9)	.096	9.1 (2.1)	9.2 (1.8)	.915
RBMT story delayed	6.0 (1.4)	7.8 (2.5)	.029^a^	9.1 (2.1)	9.3 (1.6)	.723
GDS	2.3 (1.7)	2.2 (1.7)	.876	1.3 (1.7)	1 (2.5)	.533
CDT	8.6 (1.2)	8.3 (1.2)	.287	9.8 (0.6)	9.8 (0.5)	.333
MSEQ % pictures	50.4 (15.3)	51.9 (17.7)	.791	54.7 (11.3)	66.4 (9.6)	.004^a^
MSEQ % stories	61.9 (22.6)	66.6 (17.3)	.385	76 (14.8)	66.4 (9.6)	.032^a^

* p value refers to t Test for paired samples.

a p value <0.05 1; MMSE: Mini Mental State Exam, MAC-Q: Memory Complaint Questionnaire; RBMT: Rivermead Behavioral Memory Test; GDS: Geriatric Depression Scale; CDT: Clock Drawing Test; MSEQ: Memory Self Efficacy Questionnaire.

Comparisons between post test 2008 and pre-test 2010 showed no statistically significant difference for any of the variables, indicating that participants returning for the second training intervention had a similar performance on the 2010 pre-test to that of the 2008 post-test. This finding could be associated to maintenance of the gains obtained in the first intervention or due to the retest effect.

The results also revealed a statistically significant difference for a greater number of variables between the 2010 pre and post test versus difference observed in the 2008 assessment. A significant improvement on the MMSE, MAC-Q, Incidental Memory, Delayed Memory, MSEQ pictures was found. A marginally significant difference was also observed for the Immediate Memory variable on the 2010 assessments.

Moreover, further gains were observed for the variable Incidental Memory and Delayed Memory, since improvements were seen between the 2008 and 2010 assessments.

Unexpectedly, a statistically significant change in MSEQ Stories was also seen. Gains were evident on this questionnaire after the first intervention. On the 2010 pre test, participants demonstrated greater self-efficacy for memorization of stories compared to 2008. A decline in this variable occurred on the 2010 post test after the second training intervention. However, compared to 2008 data, the MSEQ Stories results were maintained.

Thus, these results suggest that the first intervention held in 2008 yielded positive results and the 2010 intervention produced yet further gains, suggesting maintenance of the gains achieved in the first intervention.

## Discussion

The aim of this study was to examine the maintenance of results from a five-session cognitive training intervention and the benefits of booster training. A stable cognitive performance was observed between training programs and additional gains were seen in measures of episodic memory after the second training program. On the 2010 intervention, significant changes were observed in scores on the MMSE, MAC-Q, on Incidental Memory, Delayed Memory, MSEQ Pictures and on MSEQ Stories, as well as marginally significant changes in Immediate Memory. Furthermore, additional gains were found for the variables Incidental Memory and Delayed Memory.

Our data are in line with a study reporting that, six months after a training intervention, 17.9% of participants who had received booster training and were reassessed, maintained their initial performance while 66.7% achieved even greater performance than on the first intervention. Only 15.4% showed a worse performance on the second intervention compared to the first intervention.^22^

Studies have shown that eight months after completing a cognitive training program, older adults were retrained and retested, and found to have maintained gains in the second pre-test.^23^ Other follow-up studies have indicated that cognitive gains were maintained in older adults after a six-month period^24^ and longer periods of 12 months^25^ and even five years after the first intervention.^26^

Gains achieved and maintained following cognitive training could be explained by cognitive plasticity in older adults, including in the older old.^11,27^ Several mechanisms may explain the positive outcomes after training, such as increased speed of cognitive processing, use of strategies taught, and also greater attention.

In the present study, participants were found to have fewer complaints after the first intervention, and fewer still after the second intervention. This finding is in accordance with results of studies reporting that cognitive training may improve self-efficacy of participants, rendering them more confident and less prone to complaining with regard to their memory.^28^ The literature also indicates that after cognitive training, participants were more satisfied with their memory, had fewer complaints about their memory, and made greater use of memorizing strategies compared to controls.^29^

No statistically significant difference in depressive symptoms was detected in the results described. However, the raw data suggests that participants had fewer depressive symptoms since the 2008 pre test up to the 2010 post test. This result is congruent with a study which reported that, after cognitive training, participants presented reduced depressive symptoms.^30^ This decline in depressive symptoms could be related, among other factors, to an increase in the social support network which the cognitive intervention allowed or to gains in sense of self-efficacy.

Therefore, the findings of the present study indicate that it is productive to offer cognitive training to older adults,^23^ because these training sessions seem to improve not only performance on episodic memory tasks,^11,14^ but also aspects of metamemory.^7^

Nevertheless, it should be emphasized that the gains observed could have been associated to the retest effect on instruments such as the MMSE, due to the consecutive filling out of the instrument, which does not have alternative versions available. In addition, the absence of a control group for the second intervention represents a limitation of this study. Also, it is noteworthy that no control group was possible because the cognitive training originally took place within a University open to the third age, and the 2008 control group received training after the completion of the initial study, thereafter precluding the use of this group as a control.

Several factors could also be associated to the results observed, such as participant profile, where the majority were involved in other activities at the University Open to the Third Age or at other centers for the third age, keeping them physically and mentally active. Such outside interventions are difficult to control for. In addition, the mean schooling of the sample was high compared to the norm for the Brazilian population.

In view of the results and limitations of the present study, future studies should monitor the benefits of cognitive training with a control group, involve a greater number of sessions and participants, and include a longer follow-up period. Notwithstanding its limitations, the present study contributes to the literature as it is the first to test the impact of booster training sessions in Brazil.
